# Glomerular hyperfiltration is associated with dementia: A nationwide population-based study

**DOI:** 10.1371/journal.pone.0228361

**Published:** 2020-01-28

**Authors:** Min Woo Kang, Sehoon Park, Soojin Lee, Yeonhee Lee, Semin Cho, Kyungdo Han, Hanna Cho, Yaerim Kim, Yong Chul Kim, Seung Seok Han, Hajeong Lee, Jung Pyo Lee, Kwon Wook Joo, Chun Soo Lim, Yon Su Kim, Dong Ki Kim

**Affiliations:** 1 Department of Internal Medicine, Seoul National University Hospital, Seoul, Korea; 2 Department of Internal Medicine, Seoul National University College of Medicine, Seoul, Korea; 3 Department of Biomedical Sciences, Seoul National University College of Medicine, Seoul, Korea; 4 Department of Medical Statistics, College of Medicine, Catholic University of Korea, Seoul, Korea; 5 Department of Neurology, Gangnam Severance Hospital, Yonsei University College of Medicine, Seoul, Korea; 6 Department of Internal Medicine, Keimyung School of Medicine, Seoul, Korea; 7 Kidney Research Institute, Seoul National University College of Medicine, Seoul, Korea; 8 Department of Internal Medicine, Seoul National University Boramae Medical Center, Seoul, Korea; Istituto Di Ricerche Farmacologiche Mario Negri, ITALY

## Abstract

**Background:**

Glomerular hyperfiltration may be a clinical phenotype of endothelial dysfunction. Endothelial dysfunction may cause vascular dementia through the deterioration of cerebral blood flow. We aimed to identify the risk of dementia in people with glomerular hyperfiltration.

**Methods:**

Using the Korean National Health Information Database, we included subjects aged ≥45 years who underwent national health screening examinations between 2012 and 2015 and who had no previous history of end-stage renal disease or dementia (n = 2,244,582). The primary exposure was glomerular hyperfiltration. We divided the subjects into groups by sex and five-year age intervals and categorized each group into 8 intervals according to estimated glomerular filtration (eGFR). The subjects with an eGFR ≥95^th^ percentile in each group were defined as the hyperfiltration group. The outcomes were development of all types of dementia, Alzheimer's dementia and vascular dementia. Multivariable Cox proportional hazards models were used to analyze the hazard ratios (HRs) for outcomes.

**Results:**

The Hyperfiltration group showed a higher risk for the development of all types of dementia [adjusted HR 1.09 (95% CI, 1.03–1.15)] and vascular dementia [adjusted HR 1.33 (95% CI, 1.14–1.55)] than the reference group. However, the association between hyperfiltration and Alzheimer's dementia was not statistically significant.

**Conclusions:**

Glomerular hyperfiltration may be associated with dementia. In this respect, subjects with glomerular hyperfiltration should be monitored more closely for signs and symptoms of dementia.

## Introduction

Dementia is a common but devastating disease with a very large burden on patients, caregivers, and society as a whole. Dementia affected more than 47 million patients in 2015 worldwide, and the number of patients is predicted to be approximately 135 million in 2050 [1]. Given that there is no specific treatment for advanced dementia, the identification of high-risk patients and the management of their risk factors are crucial for reducing the burden of the disease [2,3].

The risk of cognitive impairment and dementia in patients with kidney dysfunction is higher than that in the general population with normal kidney function [4–8]. In this regard, vascular damage through endothelial dysfunction has explained the association of a decline in kidney function with an increased risk of dementia [8]. The prevalence of cognitive impairment is higher in patients with mild to moderate chronic kidney disease (CKD) than in those with normal kidney function and much more elevated in patients with end-stage renal disease (ESRD) [5,7,8]. Interestingly, previous studies have shown that glomerular hyperfiltration, as well as a decline in glomerular filtration rate (GFR), is associated with cardiovascular morbidity and mortality [9–15]. In this regard, glomerular hyperfiltration may be one of the clinical phenotypes of endothelial dysfunction [16,17]. Since decreased cerebrovascular reactivity and increased blood vessel tortuosity as a result of endothelial dysfunction are essential pathophysiological components of cognitive dysfunction and dementia [18], dementia might share its pathophysiology with glomerular hyperfiltration in terms of endothelial dysfunction [19,20].

In the present study, we aimed to evaluate the association between glomerular hyperfiltration and dementia using data from a nationwide population-based cohort as an effort to identify people at high risk for dementia. The risks of Alzheimer’s and vascular dementia were analyzed separately because of their different pathophysiology and management strategies [19].

## Materials and methods

### Data source

We obtained and analyzed data from the Korean National Health Information Database (NHID) from the Korean National Health Insurance System (NHIS), which is a public data resource that includes data from the whole population of South Korea. Since the NHID includes insured medical services, health screenings, and sociodemographic variables, we could review the diagnostic codes, admission history, demographics and laboratory data. The NHIS provides this charge-free health screening for workplace subscribers and for every Korean aged ≥40 years old at least biannually. This health screening is provided for approximately 15 million people every year, and the total examination rate has been consistently higher than 70% since 2011 [20].

### Ethical approval

The present study received full approval from the Institutional Review Board of Seoul National University Hospital (IRB No. 1804-023-934). The government approved access to the NHIS database for this study. Informed consent was waived because the present study retrospectively used the data provided by NHIS, and it did not perform any additional medical intervention. All authors followed the latest version of the declaration of Helsinki throughout the study.

### Study population

We included subjects aged ≥45 years who had two or more national health screening examinations between Jan 2012 and Dec 2015. Those who had end-stage renal disease (ESRD) or dementia before participating in the national health screening examinations were excluded. ESRD was defined as the commencement of dialysis or receiving kidney transplantation. The study subjects were divided based on five-year age intervals in both sexes. We assessed the estimated GFR (eGFR) distribution in each of the groups and calculated the eGFR values corresponding to the 5^th^, 20^th^, 35^th^, 50^th^, 65^th^, 80^th^, and 95^th^ percentiles. We categorized the groups divided by sex and five-year age intervals into eGFR percentile groups of <5^th^, 5^th^ -19^th^, 20^th^ -34^th^, 35^th^ -49^th^, 50^th^ -64^th^, 65^th^ -79^th^, 80^th^ -94^th^ and ≥95^th^ percentiles. eGFR was calculated using the Chronic Kidney Disease-Epidemiology Collaboration (CKD-EPI) equation [21]. The reference group was defined as the subjects with eGFR levels between the 50^th^ and 64^th^ percentile in each group. Hyperfiltration was defined as eGFR ≥95^th^ percentile in each group.

### Study outcomes

The primary outcome was the development of all types of dementia, which included vascular dementia, Alzheimer’s dementia, and other kinds of dementia. The definitions of Alzheimer’s and vascular dementia were based on the recording of International Classification of Diseases (ICD)-10 codes [22] and the prescription of medications for dementia, which were rivastigmine, galantamine, memantine, or donepezil. According to ICD-10 codes, the recording of F00 (dementia in Alzheimer’s disease), F01 (vascular dementia), F02 (dementia in other diseases classified elsewhere), F03 (unspecified dementia), G30 (Alzheimer’s disease) or G31 (other degenerative diseases of nervous system, not elsewhere classified) was defined as all types of dementia, F00 or G30 was defined as Alzheimer’s disease and F01 was defined as vascular dementia. If the subjects had codes for both Alzheimer's dementia and vascular dementia, we chose the principal diagnosis.

### Data collection

Data including age, sex, body mass index, smoking, alcohol consumption, exercise, income, diabetes mellitus, hyperlipidemia, and hypertension were collected. Smoking history was categorized into a current smoker, ex-smoker and never smoker. Alcohol consumption history was categorized into heavy drinking, mild drinking, and nondrinking. The definition of heavy drinking was a daily alcohol consumption of 30 g/day or more, and the definition of mild drinking was a daily alcohol consumption below 30 g/day. People included in the lowest quartile of the required insurance fees or receiving free insurance were categorized as the low-income group.

### Statistical analysis

Mean (± standard deviation) is used to describe continuous variables. Data are presented as percentages for categorical variables. Comparisons between normally distributed continuous variables were performed using analysis of variance. When comparing categorical variables, the chi-squared test was used. We used the Cox proportional hazards model to calculate hazard ratios (HRs) for the occurrence of all types of dementia, vascular dementia and Alzheimer’s dementia separately within the study groups. We analyzed the cubic spline regression model, setting the 60^th^ percentile of eGFR as the reference for further investigation of the relationship between GFR percentile and the development of dementia. All the variables including age, sex, body mass index, smoking history, alcohol consumption history, exercise, income, diabetes mellitus, hypertension and hyperlipidemia were adjusted in multivariable analyses. We performed a statistical analysis using the SAS 9.4 program (SAS Institute, United States). A *P* value less than 0.05 was considered statistically significant.

## Results

### Study subjects

**Fig 1:**
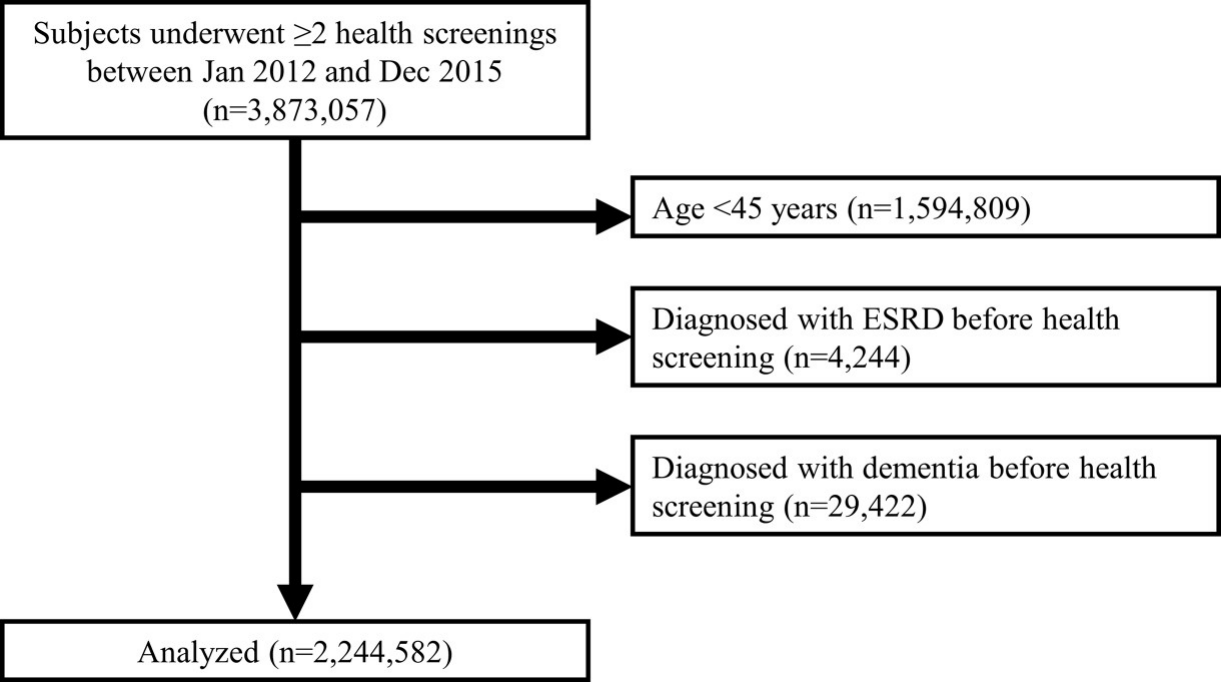
Diagram showing the study population.

**Fig 2:**
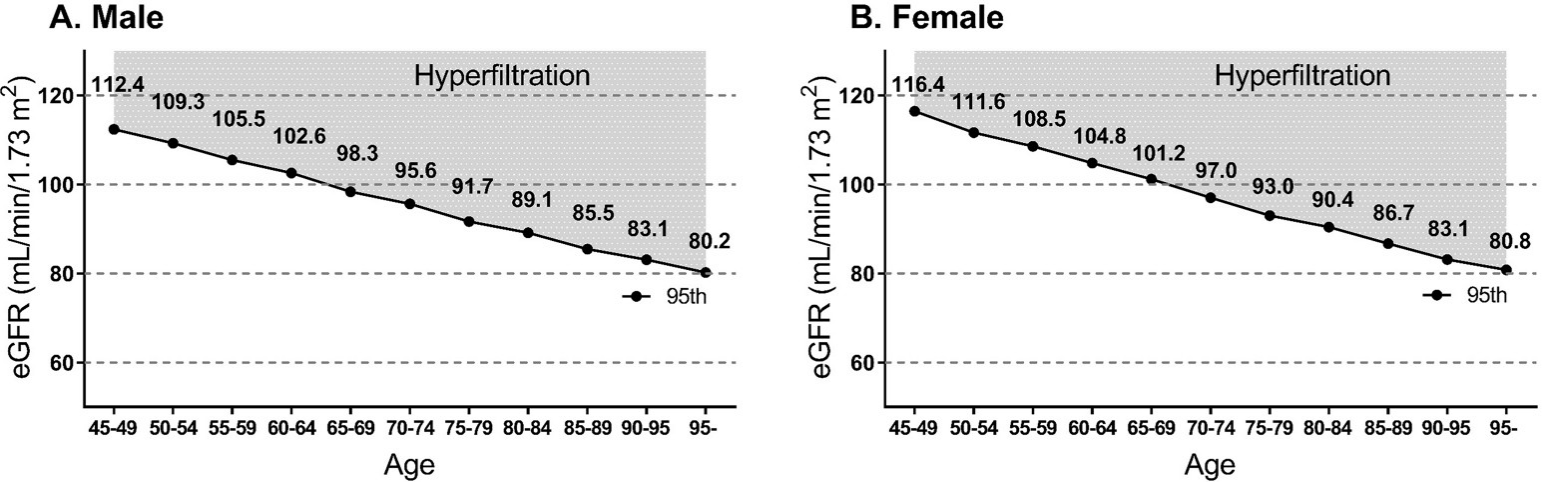
Distribution of eGFR corresponding to the definition of hyperfiltration according to age in males (A) and females (B).

There were 2,278,248 health examinees aged 45 years or older who underwent ≥2 national health screenings between Jan 2012 and Dec 2015. Subjects with ESRD (n = 4,244) or dementia (n = 29,422) before the first health screening during the study period were excluded. Therefore, 2,244,582 subjects were included in the study (Fig 1). Among the subjects, 58,624 were categorized into the hyperfiltration group. The cutoff eGFR values for hyperfiltration tended to decrease as age increased (Fig 2).

### Baseline characteristics

Baseline characteristics are shown in Table 1. Among the 8 eGFR percentile interval groups, the hyperfiltration group had the oldest age and the highest proportions of both current smokers and heavy drinkers. The hyperfiltration group had the lowest BMI. The mean eGFR value of the hyperfiltration group was 110.8 mL/min/1.73 m^2^.

**Table 1:** Baseline characteristics of the study population.

		eGFR percentile group								
Baseline characteristics	Total (n = 2,244,582)	<5 (n = 105,556)	5–19 (n = 340,776)	20–34 (n = 343,651)	35–49 (n = 205,335)	50–64 (n = 542,978)	65–79 (n = 360,267)	80–94 (n = 287,395)	95≤ (n = 58,624)	p-value
Age (years old)	58.5±9.6	59.4±9.4	58.2±9.6	59.2±9.1	58.3±12.2	58.0±8.5	58.7±8.7	58.4±10.5	60.5±11.1	< .0001
Male (%)	48.2	46.5	59.2	31.0	74.3	47.1	32.7	57.6	57.2	< .0001
Smoker										< .0001
Never smoker (%)	64.8	67.1	58.3	77.1	49.2	65.0	74.8	57.0	57.4	
Ex-smoker (%)	17.6	18.1	22.0	12.2	26.2	17.2	13.0	19.2	17.7	
Current smoker (%)	17.6	14.8	19.8	10.7	24.6	17.8	12.3	23.8	25.0	
Drinker										< .0001
Nondrinker (%)	61.3	67.8	58.2	70.5	49.5	61.0	66.9	54.8	55.8	
Mild drinker (%)	32.2	27.8	34.9	25.7	41.6	32.4	28.2	35.9	33.8	
Heavy drinker (%)	6.5	4.5	6.9	3.8	8.8	6.6	4.9	9.3	10.3	
Exercise (%)	22.1	22.0	22.8	21.9	22.8	22.4	21.8	21.0	20.0	< .0001
Low income (%)	21.8	24.3	20.4	23.6	18.0	21.6	24.0	20.6	22.2	< .0001
BMI (kg/m^2^)	24.1±3.1	24.6±3.2	24.5±3.0	24.1±3.1	24.2±3.0	24.1±3.1	23.8±3.1	23.8±3.1	23.5±3.3	< .0001
DM (%)	15.1	24.5	16.2	13.8	14.7	13.8	13.6	15.7	18.7	< .0001
HTN (%)	40.4	55.1	43.3	39.8	40.4	38.1	37.5	39.9	44.6	< .0001
Hyperlipidemia (%)	32.2	43.7	35.1	34.2	27.6	31.6	30.9	28.6	28.9	< .0001
Creatinine (mg/dL)	0.85±0.33	1.49±1.07	1.06±0.13	0.88±0.11	0.94±0.11	0.80±0.10	0.67±0.10	0.65±0.12	0.52±0.10	< .0001
eGFR (mL/min/1.73㎡)	88.1±16.1	52.6±13.7	69.7±7.8	80.0±7.6	84.4±10.3	94.9±6.0	99.5±6.9	103.1±8.2	110.8±10.2	< .0001

BMI, body mass index; DM, diabetes mellitus; HTN, hypertension; eGFR, estimated glomerular filtration rate

### Risk of all dementia types, Alzheimer’s dementia and vascular dementia

**Fig 3:**
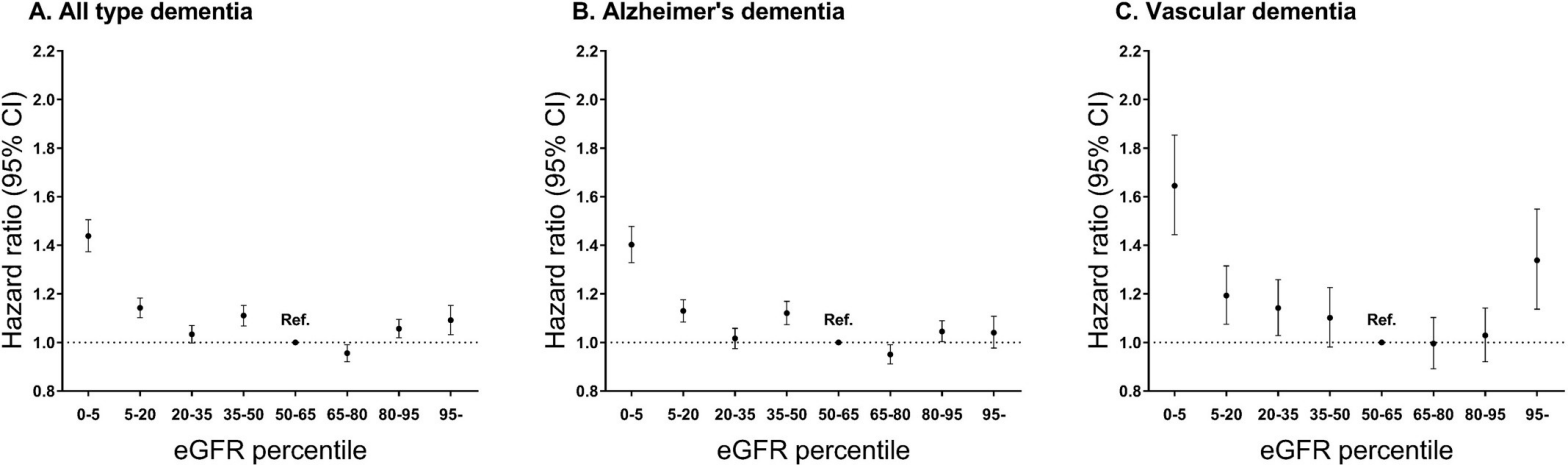
The hazard ratios of all types of dementia (A), Alzheimer's (B) and vascular dementia (C) according to eGFR.

**Fig 4:**
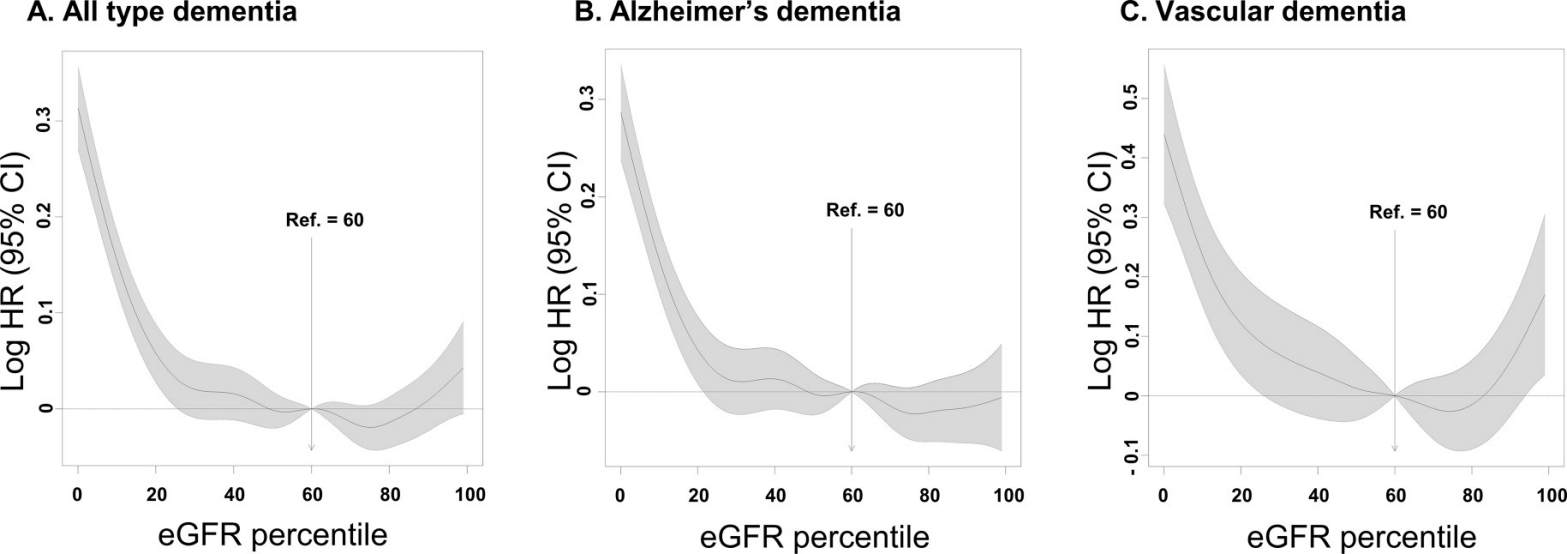
Spline curves for hazard ratios of all types of dementia (A), Alzheimer's (B) and vascular dementia (C) according to eGFR.

A total of 37,513 (1.67%) out of 2,244,582 subjects developed dementia during the study period [median follow-up duration: 3.13 (interquartile range: 2.01–4.08) years]. Alzheimer's and vascular dementia accounted for 77.3% (28,991) and 12.1% (4,551) of all types of dementia, respectively. In the hyperfiltration group, 1,596 (2.72%) subjects developed dementia. The proportions of Alzheimer's and vascular dementia in all types of dementia were similar to that in the total population (Table 2). After adjustment for age, sex, body mass index, smoking, alcohol, exercise, income, diabetes mellitus, hypertension and hyperlipidemia, the hyperfiltration group showed a higher risk of all types of dementia [adjusted hazard ratio (HR) 1.09 (95% CI, 1.03–1.15)] than the reference group. A statistically significant association was identified only for vascular dementia [adjusted HR 1.33 (95% CI, 1.14–1.55)], not in Alzheimer's dementia [adjusted HR 1.04 (95% CI, 0.98–1.11)] (Fig 3 and S1 Table). Fig 4 shows cubic spline curves and 95% confidence intervals adjusted for multivariable covariates. The HRs of both all types of dementia and vascular dementia according to the eGFR percentile tended to be U-shaped. However, Alzheimer's dementia did not show this tendency (Fig 4).

**Table 2:** The incidence of all types of dementia, Alzheimer's and vascular dementia according to eGFR percentile groups.

eGFR percentile group	Total (n = 2,244,582)	<5 (n = 105,556)	5–19 (n = 340,776)	20–34 (n = 343,651)	35–49 (n = 205,335)	50–64 (n = 542,978)	65–79 (n = 360,267)	80–94 (n = 287,395)	95≤ (n = 58,624)
All types of Dementia (n, %)	37,513 (1.67)	2,594 (2.46)	5,890 (1.73)	5,711 (1.66)	4,751 (2.31)	6,517 (1.20)	5,048 (1.40)	5,406 (1.88)	1,596 (2.72)
Alzheimer's dementia (n, %)	28,991 (1.29)	1,944 (1.84)	4,518 (1.33)	4,388 (1.28)	3,762 (1.83)	5,030 (0.93)	3,933 (1.09)	4,200 (1.46)	1,216 (2.07)
Vascular dementia (n, %)	4,551 (0.20)	370 (0.35)	737 (0.22)	723 (0.21)	532 (0.26)	795 (0.15)	599 (0.17)	591 (0.21)	204 (0.35)

eGFR = estimated glomerular filtration rate

**Fig 5:**
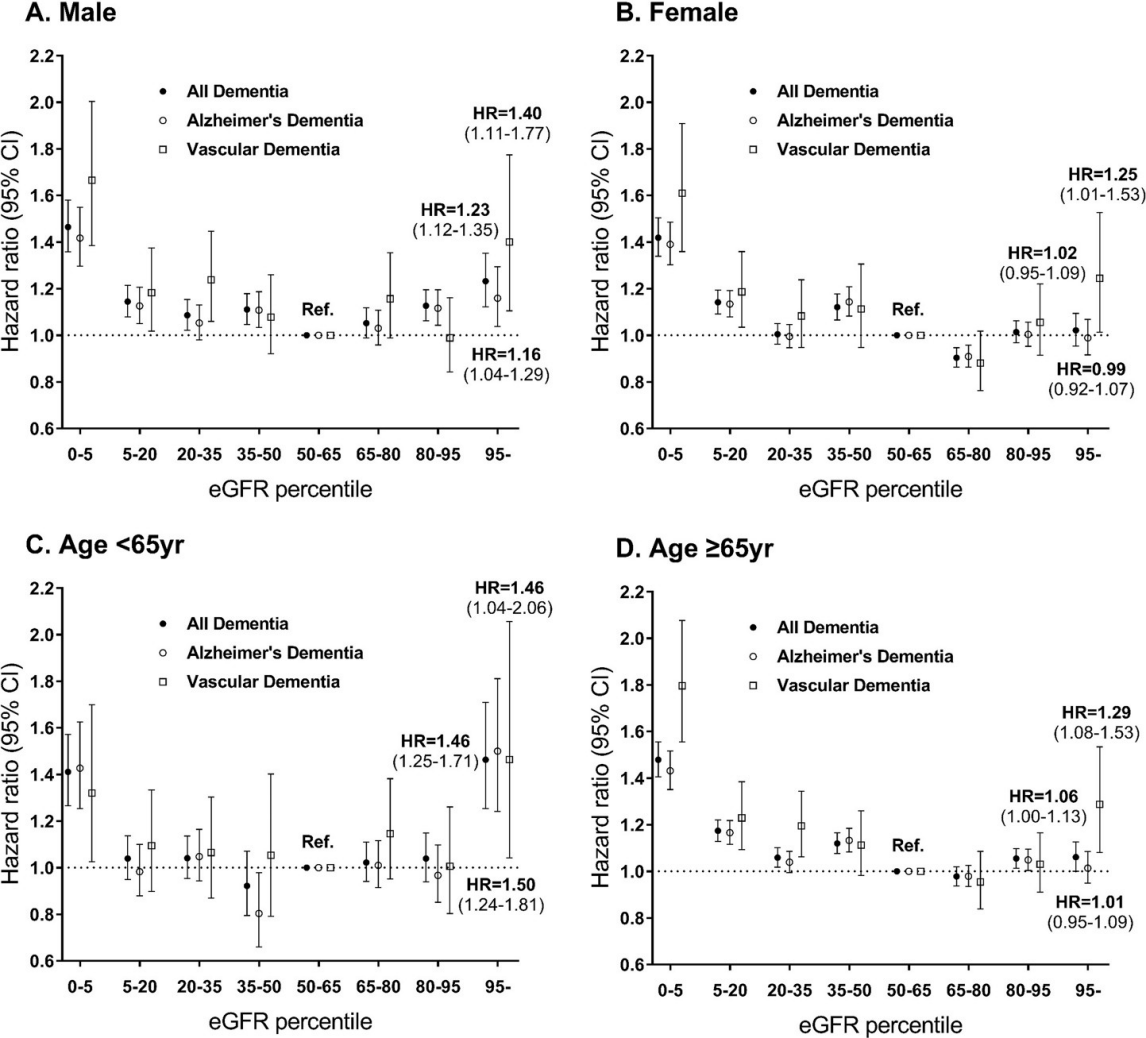
The hazard ratios of all types of dementia, Alzheimer's, and vascular dementia according to eGFR in males (A), females (B), individuals aged < 65 years (C) and individuals aged ≥ 65 years (D).

We further conducted a subgroup analysis by dividing the patients by sex. In males, the hyperfiltration group had a higher risk of all types of dementia [adjusted HR 1.23 (95% CI, 1.12–1.35); p for interaction<0.01]. When analyzing Alzheimer's and vascular dementia separately in males, the hyperfiltration groups showed a significantly increased risk of both Alzheimer's dementia [adjusted HR 1.16 (95% CI, 1.04–1.29); p for interaction<0.01] and vascular dementia [adjusted HR 1.40 (95% CI, 1.11–1.77); p for interaction = 0.06] with the same pattern for all types of dementia. In comparison, the hyperfiltration group did not show a significantly increased risk of all types of dementia [adjusted HR 1.02 (95% CI, 0.95–1.09)] in females. In females, the hyperfiltration group had a higher risk of only vascular dementia [adjusted HR 1.25 (95% CI, 1.01–1.53)] but not Alzheimer’s dementia [adjusted HR 0.99 (95% CI, 0.92–1.07)] (Fig 5 and S2 Table).

In subjects aged ≥ 65 years, the hyperfiltration group had a higher risk of all types of dementia [adjusted HR 1.06 (95% CI, 1.00–1.13)] and vascular dementia [adjusted HR 1.29 (95% CI, 1.08–1.53)] compared with the risks of the reference group. In subjects with age < 65 years, the hyperfiltration group had a higher risk of all types of dementia [adjusted HR 1.46 (95% CI, 1.25–1.71); p for interaction<0.01], Alzheimer’s dementia [adjusted HR 1.50 (95% CI, 1.24–1.81); p for interaction<0.01] and vascular dementia [adjusted HR 1.46 (95% CI, 1.04–2.06); p for interaction = 0.16] than the reference group. In subjects aged ≥ 65 years, the hyperfiltration group had a higher risk of all types of dementia [adjusted HR 1.06 (95% CI, 1.00–1.13)] and vascular dementia [adjusted HR 1.29 (95% CI, 1.08–1.53)] than the reference group (Fig 5 and S3 Table).

## Discussion

In this nationwide population-based study including 2.2 million people, we identified a significantly high risk of vascular dementia, but not Alzheimer’s dementia, in subjects with glomerular hyperfiltration. The result was statistically significant even after adjusting for well-known risk factors for vascular dementia, including diabetes mellitus, hypertension, and smoking [23–26]. Vascular dementia showed a U-shaped risk according to the GFR percentile. Subgroup analysis showed that the patterns of vascular dementia did not differ by age or sex.

Glomerular hyperfiltration is associated with various clinical outcomes, including cardiovascular events [9–15]. In a cohort of Turkish adults, the subjects with glomerular hyperfiltration, which was defined as the highest eGFR quartile, showed a 6-fold relative risk of death and cardiopulmonary events when compared to the risk of subjects with normal eGFR [11]. Similarly, glomerular hyperfiltration, which was defined as GFR>95^th^ percentile, was associated with a significantly higher risk of cardiovascular death even after adjustment for multiple risk factors such as age, sex, muscle mass, diabetes and hypertension in an Asian cohort of a general population [10]. Previous studies have suggested a J-shaped or U-shaped association between GFR and all-cause or cardiovascular mortality, which is a similar pattern of association between eGFR and dementia in this study [10,27–31]. The pathophysiological mechanisms of hyperfiltration have not been well identified. Various hormonal factors, including the renin-angiotensin system and cyclooxygenase-2, have been suggested to contribute to the development of hyperfiltration [32,33]. Furthermore, hyperfiltration has also been shown to be associated with endothelial dysfunction in several clinical conditions [16,17,34,35]. In previous studies, the association between increased risk of cardiovascular events and hyperfiltration was explained by endothelial dysfunction and arterial stiffness [9,16]. Hyperfiltration may be associated with the risk of impaired ability to induce arterial vasodilation after an ischemic stimulus and reflect general endothelial dysfunction [16]. Hyperfiltration was also associated with a paradoxical state of high renal and low systemic vascular nitric oxide (NO) bioactivity [17]. Overall, glomerular hyperfiltration may be associated with vascular damage through which cardiovascular and kidney disease can be potentially influenced [9,36,37]. Hypoxia caused by cerebral blood flow deterioration is an important cause of vascular dementia [38]. Because of the impaired ability to induce arterial vasodilation after an ischemic stimulus and low systemic NO bioactivity, the risk of hypoxia may be higher in patients with hyperfiltration than in healthy people, resulting in a high risk of vascular dementia. Though the association between hyperfiltration and endothelial dysfunction has been found mostly in diabetic patients, hyperfiltration was also identified to be associated with subclinical vascular damage in patients without diabetes mellitus [39]. Vascular damage potentially associated with glomerular hyperfiltration may contribute to the development of vascular dementia. However, further studies are needed to identify the mechanisms by which hyperfiltration is associated with dementia.

In the present study, hyperfiltration groups among males or individuals less than 65 years old showed a higher risk of Alzheimer's dementia than the reference group. Vascular damage is one of the mechanisms for the development of Alzheimer's dementia [40,41]. Therefore, we thought that Alzheimer's dementia could also show U-shaped risk according to eGFR. However, the incidence rate of Alzheimer's dementia was higher in women than in men [42,43], indicating that there may be differences in the mechanisms of the development of Alzheimer's dementia between males and females [44,45]. Additionally, the incidence rate of Alzheimer’s dementia differs by age [46–48]. Therefore, we thought that the reason why Alzheimer's dementia did not show U-shaped risk was that the mechanism for the development of Alzheimer's dementia might be different in some degree by sex and age. However, further study is needed to identify the exact cause and pathophysiology.

No single definition of glomerular hyperfiltration has been agreed upon [35]. Conventionally, a range of eGFR, which is over two standard deviations above the mean GFR of healthy individuals, has been used as the definition of glomerular hyperfiltration. Creatinine clearance declines with age [49]. GFR has been known to decrease by 1.05 ml/min/year in the aged 70–110 years old [50]. Some studies have defined hyperfiltration with an absolute eGFR value without considering the age-dependent decline in GFR [51,52]. This could cause normal GFR in young subjects to be misclassified as hyperfiltration. In addition, the decline in GFR with age is known to vary by sex [53]. A previous systematic review suggested that the glomerular hyperfiltration threshold should be adjusted for age and sex, especially in studies including the elderly [54]. Therefore, the present study used the 95^th^ percentile of eGFR after dividing by sex and five-year age intervals as a cutoff value, which might be more reasonable than the simple definitions of hyperfiltration used in previous studies. Adjusting the levels of eGFR corresponding to hyperfiltration for each age group might allow us to classify elderly subjects with hyperfiltration more correctively.

The strength of this study was that we studied the association of glomerular hyperfiltration with dementia in a large cohort from the NHIS, which covers all people with South Korean nationality. In addition, we investigated the risk of Alzheimer’s dementia and vascular dementia separately, which would further enhance the comprehensibility of our study results.

There are several limitations in this study. First, muscle mass was not considered in the definition of hyperfiltration. The overestimation of true GFR by eGFR based on serum creatinine level in subjects with decreased muscle mass could result in misclassification of hyperfiltration. Second, the definition of dementia was defined by diagnostic codes not using cognitive function tests or other modalities. Patients whose diagnosis had changed over the follow-up duration could be misclassified. Third, as we used creatinine measured once at the time when follow-up started to calculate eGFR, transient renal function changes may have caused the misclassification of the hyperfiltration group. Forth, the elderly with preserved kidney function could be misclassified into hyperfiltration. Finally, the data on the duration of diabetes mellitus and hypertension were not available in this dataset.

In conclusion, glomerular hyperfiltration may be a predictor of dementia, especially vascular dementia, and identifying individuals with hyperfiltration may be an effective preventive strategy in dementia. Healthcare providers should be aware of the potential risk of dementia in people with glomerular hyperfiltration. Whether treatments of clinical conditions causing glomerular hyperfiltration can prevent or retard the development of dementia should be studied in long-term intervention studies.

## Supporting information

S1 Table The hazard ratios of all types, Alzheimer's and vascular dementia. (DOCX)

S2 Table Hazard ratios of all types, Alzheimer's and vascular dementia according to sex. (DOCX)

S3 Table Hazard ratios of all types, Alzheimer's and vascular dementia according to age. (DOCX)
