# Supplementary material for: Glomerular hyperfiltration is associated with dementia: A nationwide population-based study
Source: PLoS One. 2020 Jan 28;15(1):e0228361. doi: 10.1371/journal.pone.0228361 (PMC6986766; doi:10.1371/journal.pone.0228361)
Supplement: S1 Table — (DOCX) [file pone.0228361.s001.docx]

S1 Table. The hazard ratios of all types, Alzheimer's and vascular dementia

|  | All types of dementia | | Alzheimer's dementia | | Vascular dementia | |
| --- | --- | --- | --- | --- | --- | --- |
| eGFR percentile group | HR (95% CI) | p-value | HR (95% CI) | p-value | HR (95% CI) | p-value |
| <5 | 1.44 (1.37-1.51) | <0.001 | 1.40 (1.33-1.48) | <0.001 | 1.64 (1.44-1.85) | <0.001 |
| 5-19 | 1.14 (1.10-1.18) | <0.001 | 1.13 (1.08-1.18) | <0.001 | 1.19 (1.08-1.32) | <0.001 |
| 20-34 | 1.03 (1.00-1.07) | 0.076 | 1.02 (0.98-1.06) | 0.457 | 1.14 (1.03-1.26) | 0.012 |
| 35-49 | 1.11 (1.07-1.15) | <0.001 | 1.12 (1.07-1.17) | <0.001 | 1.10 (0.98-1.23) | 0.104 |
| 50-64 | (reference) |  | (reference) |  | (reference) |  |
| 65-79 | 0.96 (0.92-0.99) | 0.015 | 0.95 (0.91-0.99) | 0.016 | 0.99 (0.89-1.10) | 0.885 |
| 80-94 | 1.06 (1.02-1.10) | 0.003 | 1.05 (1.00-1.09) | 0.037 | 1.03 (0.92-1.14) | 0.653 |
| 95≤ | 1.09 (1.03-1.15) | 0.002 | 1.04 (0.98-1.11) | 0.221 | 1.33 (1.14-1.55) | <0.001 |

eGFR, estimated glomerular filtration rate; HR, hazard ratio; CI, confidence interval
