# Supplementary material for: Glomerular hyperfiltration is associated with dementia: A nationwide population-based study
Source: PLoS One. 2020 Jan 28;15(1):e0228361. doi: 10.1371/journal.pone.0228361 (PMC6986766; doi:10.1371/journal.pone.0228361)
Supplement: S2 Table — (DOCX) [file pone.0228361.s002.docx]

S2 Table. Hazard ratios of all types, Alzheimer's and vascular dementia according to sex.

|  |  | | | All types of dementia | | | Alzheimer's dementia | | | | Vascular dementia | | | |
| --- | --- | --- | --- | --- | --- | --- | --- | --- | --- | --- | --- | --- | --- | --- |
| Sex | | eGFR percentile group | HR (95% CI) | | p-value | p for interaction | | HR (95% CI) | p-value | p for interaction | | HR (95% CI) | p-value | p for interaction |
| Male | | <5 | 1.47 (1.36-1.58) | | <0.001 |  | | 1.42 (1.30-1.55) | <0.001 |  | | 1.67 (1.39-2.00) | <0.001 |  |
|  |  | 5-19 | 1.15 (1.08-1.22) | | <0.001 |  | | 1.13 (1.05-1.21) | <0.001 |  | | 1.18 (1.02-1.38) | 0.028 |  |
|  |  | 20-34 | 1.09 (1.02-1.15) | | 0.008 |  | | 1.05 (0.98-1.13) | 0.158 |  | | 1.24 (1.06-1.45) | 0.007 |  |
|  |  | 35-49 | 1.11 (1.05-1.18) | | 0.001 |  | | 1.11 (1.03-1.19) | 0.004 |  | | 1.08 (0.92-1.26) | 0.350 |  |
|  |  | 50-64 | (reference) | |  |  | | (reference) |  |  | | (reference) |  |  |
|  |  | 65-79 | 1.05 (0.99-1.12) | | 0.107 |  | | 1.03 (0.96-1.11) | 0.413 |  | | 1.16 (0.99-1.35) | 0.069 |  |
|  |  | 80-94 | 1.13 (1.06-1.20) | | <0.001 |  | | 1.12 (1.04-1.20) | 0.002 |  | | 0.99 (0.84-1.16) | 0.896 |  |
|  |  | 95≤ | 1.23 (1.12-1.35) | | <0.001 | <0.001 | | 1.16 (1.04-1.29) | 0.009 | <0.001 | | 1.40 (1.11-1.77) | 0.005 | 0.058 |
| Female | | <5 | 1.42 (1.34-1.50) | | <0.001 |  | | 1.39 (1.30-1.49) | <0.001 |  | | 1.61 (1.36-1.91) | <0.001 |  |
|  |  | 5-19 | 1.14 (1.09-1.19) | | <0.001 |  | | 1.13 (1.08-1.19) | <0.001 |  | | 1.19 (1.04-1.36) | 0.014 |  |
|  |  | 20-34 | 1.01 (0.96-1.05) | | 0.814 |  | | 1.00 (0.95-1.05) | <0.001 |  | | 1.08 (0.95-1.24) | 0.241 |  |
|  |  | 35-49 | 1.12 (1.07-1.18) | | <0.001 |  | | 1.14 (1.08-1.21) | <0.001 |  | | 1.11 (0.95-1.31) | 0.190 |  |
|  |  | 50-64 | (reference) | |  |  | | (reference) |  |  | | (reference) |  |  |
|  |  | 65-79 | 0.90 (0.86-0.95) | | <0.001 |  | | 0.91 (0.86-0.96) | <0.001 |  | | 0.88 (0.76-1.02) | 0.085 |  |
|  |  | 80-94 | 1.01 (0.97-1.06) | | 0.553 |  | | 1.00 (0.95-1.06) | 0.886 |  | | 1.06 (0.91-1.22) | 0.458 |  |
|  |  | 95≤ | 1.02 (0.95-1.09) | | 0.542 |  | | 0.99 (0.92-1.07) | 0.786 |  | | 1.25 (1.01-1.53) | 0.036 |  |

eGFR, estimated glomerular filtration rate; HR, hazard ratio; CI, confidence interval
