# Supplementary material for: Glomerular hyperfiltration is associated with dementia: A nationwide population-based study
Source: PLoS One. 2020 Jan 28;15(1):e0228361. doi: 10.1371/journal.pone.0228361 (PMC6986766; doi:10.1371/journal.pone.0228361)
Supplement: S3 Table — (DOCX) [file pone.0228361.s003.docx]

S3 Table. Hazard ratios of all types, Alzheimer's and vascular dementia according to age.

|  |  | | | All types of dementia | | | Alzheimer's dementia | | | | Vascular dementia | | | |  |
| --- | --- | --- | --- | --- | --- | --- | --- | --- | --- | --- | --- | --- | --- | --- | --- |
| Age | | eGFR percentile group | HR (95% CI) | | p-value | p for interaction | | HR (95% CI) | p-value | p for interaction | | HR (95% CI) | p-value | p for interaction | |
| < 65 | | <5 | 1.41 (1.27-1.57) | | <0.001 |  | | 1.43 (1.25-1.63) | <0.001 |  | | 1.32 (1.03-1.70) | 0.031 |  | |
|  |  | 5-19 | 1.04 (0.95-1.14) | | 0.407 |  | | 0.98 (0.88-1.10) | 0.770 |  | | 1.09 (0.90-1.33) | 0.373 |  | |
|  |  | 20-34 | 1.04 (0.95-1.14) | | 0.370 |  | | 1.05 (0.94-1.16) | 0.380 |  | | 1.07 (0.87-1.30) | 0.543 |  | |
|  |  | 35-49 | 0.92 (0.80-1.07) | | 0.285 |  | | 0.80 (0.66-0.98) | 0.030 |  | | 1.05 (0.79-1.40) | 0.719 |  | |
|  |  | 50-64 | (reference) | |  |  | | (reference) |  |  | | (reference) |  |  | |
|  |  | 65-79 | 1.02 (0.94-1.11) | | 0.607 |  | | 1.01 (0.91-1.12) | 0.849 |  | | 1.15(0.95-1.38) | 0.153 |  | |
|  |  | 80-94 | 1.04 (0.94-1.15) | | 0.461 |  | | 0.97 (0.85-1.10) | 0.600 |  | | 1.01 (0.80-1.26) | 0.959 |  | |
|  |  | 95≤ | 1.46 (1.25-1.71) | | <0.001 | <0.001 | | 1.50 (1.24-1.81) | <0.001 | <0.001 | | 1.46 (1.04-2.06) | 0.028 | 0.156 | |
| ≥ 65 | | <5 | 1.48 (1.41-1.56) | | <0.001 |  | | 1.43 (1.35-1.52) | <0.001 |  | | 1.80 (1.56-2.08) | <0.001 |  | |
|  |  | 5-19 | 1.17 (1.13-1.22) | | <0.001 |  | | 1.17 (1.12-1.22) | <0.001 |  | | 1.23 (1.09-1.38) | <0.001 |  | |
|  |  | 20-34 | 1.06 (1.02-1.10) | | 0.004 |  | | 1.04 (0.99-1.09) | 0.092 |  | | 1.20 (1.06-1.34) | 0.003 |  | |
|  |  | 35-49 | 1.12 (1.08-1.17) | | <0.001 |  | | 1.13 (1.08-1.19) | <0.001 |  | | 1.11 (0.98-1.26) | 0.093 |  | |
|  |  | 50-64 | (reference) | |  |  | | (reference) |  |  | | (reference) |  |  | |
|  |  | 65-79 | 0.98 (0.94-1.02) | | 0.280 |  | | 0.98 (0.94-1.03) | 0.368 |  | | 0.95 (0.84-1.09) | 0.477 |  | |
|  |  | 80-94 | 1.06 (1.01-1.10) | | 0.007 |  | | 1.05 (1.00-1.10) | 0.032 |  | | 1.03 (0.91-1.17) | 0.637 |  | |
|  |  | 95≤ | 1.06 (1.00-1.13) | | 0.049 |  | | 1.01 (0.95-1.09) | 0.676 |  | | 1.29 (1.08-1.53) | 0.005 |  | |

eGFR, estimated glomerular filtration rate; HR, hazard ratio; CI, confidence interval
